# Early Diagnosis of Diabetic Neuropathy: A Review of Current Diagnostic Tests

**DOI:** 10.7759/cureus.97880

**Published:** 2025-11-26

**Authors:** Minnan Al-Khafaji, Vanessa Otti

**Affiliations:** 1 Ophthalmology, The Royal London Hospital, London, GBR; 2 Biomedical Sciences, University of Exeter, Exeter, GBR

**Keywords:** corneal confocal microscopy, diabetes screening, diabetic neuropathy, diagnostic tests, distal sensorimotor polyneuropathy, early diagnosis, review

## Abstract

Diabetes mellitus is a common chronic condition frequently complicated by distal sensorimotor polyneuropathy (DSPN), a major contributor to pain, foot ulceration, and limb loss.

Despite its impact, DSPN often remains undetected until there is significant loss of protective sensation. Routine screening practices tend to identify more advanced stages of neuropathy, offering limited sensitivity for early disease. Earlier diagnosis could substantially improve outcomes by enabling timely intervention and optimised glycaemic control.

A range of diagnostic modalities has been investigated for the early detection of DSPN, particularly those assessing small fibre function. Quantitative sensory testing (QST) is non-invasive but limited by subjectivity and variability. More objective measures include skin biopsy for intraepidermal nerve fibre density (IENFD) and corneal confocal microscopy (CCM). Both techniques provide valuable structural information about small fibre integrity; however, CCM has practical advantages as it is non-invasive, repeatable, and suitable for both clinical and research settings.

Sudomotor tests such as Neuropad are simple to administer and may detect subclinical neuropathy, though specificity is modest. The absence of a universally accepted reference standard for small fibre neuropathy complicates the comparative evaluation of diagnostic tools.

This review evaluates multiple modalities that show promise for earlier detection of DSPN. Standardisation of methodology and consensus on diagnostic criteria are essential to enable the integration of these tools into clinical practice and improve long-term patient outcomes.

## Introduction and background

Diabetes mellitus (DM) represents a major global health challenge, affecting an estimated 830 million individuals worldwide as of 2022 [1]. One of its most disabling complications is diabetic neuropathy (DN), which occurs in up to 50% of patients in the United Kingdom [2] and is strongly associated with poor glycaemic control and disease duration [3]. Hyperglycaemia plays a key role in the development of DN, especially in patients with type 1 DM; however, the precise causal relationship is less clear [4]. The most common subtype, distal sensorimotor polyneuropathy (DSPN) [5], accounts for approximately 75% of diabetic neuropathies [6], affecting both type 1 and type 2 diabetes [7]. DSPN is defined as the presence of clinical symptoms and/or signs of peripheral nerve dysfunction in individuals with diabetes, after excluding alternative causes [8]. The pathogenesis is multifactorial, involving metabolic derangements, oxidative stress (an imbalance between free radicals and antioxidant defences), and inflammatory pathways driven by chronic hyperglycaemia [6], and is more common with increasing age and time since diabetes onset [9].

DSPN typically develops in a length-dependent manner, with symptoms first manifesting in the feet [7]. Reduced sensory perception predisposes individuals to unnoticed injury, foot deformities, ulceration, and ultimately amputation [10]. The five-year mortality following diabetic foot ulceration is approximately 42% [11], while survival after amputation is lower than for breast and prostate cancer, conditions with established early detection programmes [12], with mortality rates post-amputation reaching as high as 70% [13]. This is considerably higher than the mortality observed in patients with breast (10.6%) and prostate cancer (10%) [12]. The economic burden is also substantial; NHS England spent an estimated £662 million on diabetic foot care in 2010-2011 [14]. Despite this, DSPN is frequently underdiagnosed [15], particularly as up to 50% of cases are asymptomatic [9], while others may develop neuropathic pain [10], which can reduce quality of life, cause sleep disturbance, and affect physical functioning [16]. Early detection is therefore critical, both to prevent complications and to implement timely interventions that may slow progression [17].

Current UK guidelines, as defined by the National Institute for Health and Care Excellence (NICE) Quality Outcomes Framework (QOF), recommend annual foot screening using a 10 g monofilament [18]. While the test is rapid and practical, it predominantly identifies advanced disease after protective sensation has been lost [10]. Nerve conduction studies (NCS) are considered the gold standard for assessing large fibre (responsible for vibration and motor function) neuropathy [19], as they are objective, non-invasive, and reliable [7]. However, they fail to detect early small fibre (responsible for pain and temperature sensation) dysfunction [10,20]. Small fibres, consisting of Aδ and C fibres, represent approximately 70-90% of peripheral nerves [21] and are affected earliest in DSPN [22-25]. Recent electrophysiological work has reaffirmed that Aδ and C fibres are affected at an early stage in DN, underscoring the diagnostic imperative of small fibre assessment [26]. Importantly, these fibres also have the potential to repair [27], highlighting that early detection and intervention may improve patient outcomes.

This review synthesises current evidence on diagnostic modalities for the early detection of DSPN, with a particular emphasis on small fibre assessment [28]. Moreover, recent expert consensus emphasises early recognition of small fibre dysfunction in diabetes and recommends a screening and diagnostic algorithm prioritising small fibre modalities in at-risk populations [29]. The diagnosis is typically established through a combination of methods that include clinical history, symptoms, signs, quantitative sensory testing (QST), and NCS, although small fibre evaluation remains a central focus [8].

Search strategy

A comprehensive literature search was conducted to identify studies evaluating diagnostic tests and screening approaches for DN in individuals with type 1 or type 2 DM. Searches were performed in PubMed, Embase, and CINAHL from database inception to December 2024. No language restrictions were applied, although only English-language publications were included in the review.

The search strategy combined controlled vocabulary (Medical Subject Headings (MeSH) in PubMed, Emtree terms in Embase, and CINAHL Headings) and free-text keywords. The key concepts included DM (e.g., “diabetes mellitus,” “type 1 diabetes,” “type 2 diabetes”), DN (e.g., “diabetic neuropathy,” “peripheral neuropathy,” “diabetic polyneuropathy,” “autonomic neuropathy”), and diagnostic testing or screening methods (e.g., “diagnosis,” “screening,” “early detection,” “nerve conduction,” “monofilament test,” “vibration perception threshold,” “quantitative sensory testing,” “corneal confocal microscopy”). Boolean operators “AND” and “OR” were used to combine terms appropriately, with “NOT” used to exclude irrelevant studies where necessary.

Studies were included if they reported diagnostic methods for early detection of DSPN in adults with type 1 or type 2 diabetes. Excluded studies were non-English publications, conference abstracts, case reports, and studies not reporting relevant diagnostic outcomes.

The quality and potential bias of included studies were considered qualitatively based on study design, sample size, and methodology.

Given that this is a descriptive review, results were synthesised narratively without formal meta-analysis or quantitative pooling.

A summary of the complete search strategy is presented in Table 1.

**Table 1:** PRISMA-style search strategy table. Literature search strategy for studies on early diagnosis of diabetic sensorimotor polyneuropathy (DSPN). Searches were performed in PubMed, Embase, and the Cochrane Library up to June 2025, using combinations of controlled vocabulary and keywords related to “diabetic neuropathy,” “small fibre,” “diagnosis,” and “screening.” Reference lists of included articles and relevant reviews were also examined to ensure completeness. Inclusion criteria were original studies, systematic reviews, or meta-analyses reporting diagnostic methods for early DSPN. Non-English language papers, conference abstracts, and case reports were excluded. CINAHL: Cumulative Index to Nursing and Allied Health Literature; PRISMA: Preferred Reporting Items for Systematic Reviews and Meta-Analyses; DSPN: Distal Sensorimotor Polyneuropathy.

Database	Date searched	Search string
PubMed	June 6, 2025	("Diabetes Mellitus"[Mesh] OR diabetes OR "type 1 diabetes" OR "type 2 diabetes" OR diabetic) AND ("Diabetic Neuropathies"[Mesh] OR "diabetic neuropathy" OR "peripheral neuropathy" OR "diabetic polyneuropathy" OR "autonomic neuropathy") AND ("Diagnosis"[Mesh] OR "diagnostic test" OR screening OR "nerve conduction" OR "monofilament test" OR "vibration perception threshold" OR "quantitative sensory testing" OR "corneal confocal microscopy")
Embase	June 6, 2025	('diabetes mellitus'/exp OR diabetes OR 'type 1 diabetes' OR 'type 2 diabetes' OR diabetic) AND ('diabetic neuropathy'/exp OR 'peripheral neuropathy' OR 'diabetic polyneuropathy' OR 'autonomic neuropathy') AND ('diagnosis'/exp OR 'diagnostic test' OR screening OR 'nerve conduction' OR 'monofilament test' OR 'vibration perception threshold' OR 'quantitative sensory testing' OR 'corneal confocal microscopy')
CINAHL	June 6, 2025	(MH "Diabetes Mellitus*" OR diabetes OR "type 1 diabetes" OR "type 2 diabetes" OR diabetic) AND (MH "Diabetic Neuropathies*" OR "diabetic neuropathy" OR "peripheral neuropathy" OR "diabetic polyneuropathy" OR "autonomic neuropathy") AND (MH "Diagnosis*" OR "diagnostic test" OR screening OR "nerve conduction" OR "monofilament test" OR "vibration perception threshold" OR "quantitative sensory testing" OR "corneal confocal microscopy")
Google Scholar	June 6, 2025	"diabetic neuropathy" AND ("diagnostic test" OR screening OR "nerve conduction" OR "monofilament test")
OpenGrey	June 6, 2025	"diabetic neuropathy" AND (diagnostic OR screening)

Pathophysiology

Peripheral nerves are composed of several fibre classes with distinct structures and functions: large myelinated Aα and Aβ fibres responsible for motor and proprioceptive function, thinly myelinated Aδ fibres mediating temperature and nociception, and unmyelinated C fibres conveying pain and autonomic signals [15]. In DSPN, damage typically develops in a length-dependent manner, with the distal extremities affected first. Small fibre injury is thought to occur earlier than large fibre involvement, and its dysfunction underlies many of the earliest clinical manifestations of DSPN (Table 2). Despite extensive investigation, the precise mechanisms driving this neuropathy are only partially understood, particularly the distinction between painful and painless forms [30]. Nevertheless, chronic hyperglycaemia is widely recognised as a central driver, initiating multiple interconnected pathways that converge on oxidative stress, vascular insufficiency, and axonal degeneration.

**Table 2:** Peripheral nerve fibres and function. The symptoms of diabetic peripheral neuropathy relate to the type of nerve fibres involved. Sensory symptoms involving the Aβ, Aδ, and C nerve fibres can be positive (e.g., burning, tightness, tingling) or negative (e.g., numbness, often described as a sensation similar to wearing stockings or gloves). Motor symptoms involving the Aα nerve fibres include muscle weakness and difficulty climbing stairs. Autonomic symptoms involving C fibres affect the cardiovascular, gastrointestinal, and genitourinary systems, as well as the sweat glands, and may lead to abdominal pain, urinary problems, or altered sweating. DPN may affect different sets of nerves to varying degrees. Hyperalgesia refers to increased pain sensitivity to heat, cold, pin-prick stimuli, or blunt pressure; allodynia is pain in response to non-nociceptive stimuli; hypoesthesia is decreased sensitivity to non-painful stimuli; and hypoalgesia is decreased sensitivity to painful stimuli. SWMF: Semmes-Weinstein monofilament; DPN: Diabetic Peripheral Neuropathy. Adapted from reference [15].

Classification (size)	Function	Clinical sign and sensory testing
Myelinated Aα (13-20 μm)	Motor	Decreased ankle and knee reflexes
Thickly myelinated Aβ (6-12 μm)	Proprioception, vibration, pressure	Impaired proprioception; decreased vibration sense (128 Hz tuning fork); hyperalgesia (cotton swab); hypoesthesia (10-g SWMF)
Thinly myelinated Aδ fibres (1-5 μm)	Cold, sharp pain	Hyperalgesia, hypoesthesia, or hypoalgesia (Tip-Therm, AXON GmbH); allodynia; hyperalgesia (cotton swab); hypoesthesia (10-g SWMF); hypoalgesia (pin-prick)
Unmyelinated C fibres (0.2-1.5 μm)	Warmth, burning pain, autonomic function	Hyperalgesia, hypoesthesia, or hypoalgesia (Tip-Therm); hyperalgesia (cotton swab); hypoesthesia (10-g SWMF); hypoalgesia (pin-prick); decreased sweating

One of the best-characterised mechanisms is the polyol pathway. Under hyperglycaemic conditions, aldose reductase catalyses the reduction of glucose to sorbitol, which is subsequently converted to fructose [30]. This diversion of glucose metabolism increases turnover of nicotinamide adenine dinucleotide phosphate (NADPH), leading to depletion of reduced glutathione, one of the most important cellular antioxidants. The resulting imbalance promotes the accumulation of reactive oxygen species (ROS) and oxidative stress [30]. Polyol pathway hyperactivity also exacerbates the formation of advanced glycation end-products (AGEs), which are generated through non-enzymatic glycation and oxidation of proteins and lipids. AGEs contribute to neuronal and vascular dysfunction through cross-linking of structural proteins, modification of extracellular matrix components, and binding to AGE receptors, which activate pro-inflammatory cytokines and necrotic signalling cascades. These changes impair axonal transport, disrupt neuronal repair processes, and enhance vulnerability to oxidative damage [30].

Multiple metabolic and lipid-related mechanisms contribute to the pathogenesis of DSPN. Under hyperglycaemic conditions, glucose autoxidation increases oxidative stress [31]. Hyperglycaemia also impairs fatty acid metabolism, altering the lipid composition of neuronal membranes, while excess saturated fatty acids, often concurrent in hyperglycaemia, promote mitochondrial dysfunction within Schwann cells [32,33]. These metabolic disruptions compromise myelin integrity and axonal energy homeostasis, ultimately leading to axonal degeneration. In addition, circulating low-density lipoproteins (LDLs) undergo oxidative modification in the diabetic milieu. Oxidised LDLs (ox-LDLs) interact with lectin-like ox-LDL receptors (LOX-1) on neurons and glial cells, stimulating NADPH oxidase activity and generating ROS, which further exacerbates oxidative injury and propagates neuronal dysfunction [32,34].

Another pathway implicated in DSPN pathogenesis involves protein kinase C (PKC). Persistent hyperglycaemia increases intracellular diacylglycerol concentrations, which activate PKC isoforms, particularly PKC-δ and PKC-θ. PKC activation leads to a cascade of downstream effects, including altered blood flow due to endothelial dysfunction, impaired nitric oxide availability, increased vascular permeability, and inflammation. Within neural tissues, PKC contributes to apoptosis, ion channel dysregulation, and impaired neurotrophic signalling. The convergence of these effects compounds the structural and functional decline of peripheral nerves [32,35].

Collectively, these metabolic, oxidative, and inflammatory mechanisms create a vicious cycle. Excess ROS damages DNA, proteins, and lipids, which further impairs mitochondrial function and reduces adenosine triphosphate (ATP) availability. Concurrent microvascular injury leads to nerve hypoxia and reduced clearance of toxic metabolites, further aggravating axonal stress. Together, these processes not only accelerate neurodegeneration but also diminish the capacity for nerve repair, helping to explain the progressive and often irreversible course of DSPN.

Although these mechanisms have been supported by both experimental and clinical studies, therapeutic translation has been challenging. Aldose reductase inhibitors, which target the polyol pathway, demonstrated efficacy in preventing neuropathy in animal models, but clinical trials in humans have yielded inconsistent results, as summarised in a Cochrane review by Chalk C et al. [36]. Similarly, interventions aimed at reducing AGEs or PKC activation have not demonstrated significant clinical benefits. These discrepancies highlight the multifactorial nature of DSPN and the likelihood that a combined, rather than single, therapeutic approach may be necessary for meaningful disease modification.

## Review

Tests for early diagnosis of DSPN

Questionnaires

Clinical questionnaires outlined in Table 3 remain one of the most accessible methods for assessing diabetic neuropathy, though their diagnostic accuracy varies considerably. Symptom-focused instruments such as the Neuropathy Symptom Profile [37], the Diabetic Neuropathy Symptom Score (DNS) [38], and the Neuropathy Symptom Score of the Lower Limbs (NSS-LL) [39] capture patients’ subjective experiences of pain, tingling, and numbness. These tools are inexpensive and suitable for use in large populations, yet their reliance on patient recall limits their reliability, particularly for asymptomatic early-stage disease.

**Table 3:** DSPN questionnaire groupings. Summary of commonly used questionnaires for DSPN. Each tool varies in scope, reproducibility, and suitability for clinical practice or research, with most limited by low sensitivity in asymptomatic patients and in the early stages of disease. DSPN: Distal Sensorimotor Polyneuropathy.

Category	Questionnaire/Tool	Focus	Key features
Symptom-focused	Neuropathy Symptoms Profile [35]	Patient-reported symptoms	Pain, burning, numbness, tingling
	Diabetic Neuropathy Symptom Score (DNS) [36]	Diabetic neuropathy symptoms	Pain characteristics, sensory disturbances
	Neuropathy Symptom Score of the Lower Limbs (NSS-LL) [37]	Lower extremity symptoms	Foot symptoms, mobility issues
Examination-based	Neuropathy Disability Score (NDS) [38]	Clinical examination findings	Vibration, temperature, pinprick, reflexes
	Toronto Clinical Neuropathy Score (TCNS) [39]	Combines symptoms and signs	Sensory tests, reflexes, vibration perception
Comprehensive hybrid	Michigan Neuropathy Screening Test [40]	Screening tool	Subjective and objective components
	Michigan Neuropathy Screening Instrument (MNSI) [40]	Screening with exam component	Symptom questionnaire and physical examination
Disability / function-oriented	Neuropathy Disability Score* [38]	Functional limitations	Impact on daily activities and quality of life
	Neuropathy Impairment Score (NIS) [41]	Neurological impairments	Detailed multi-domain assessment
Early detection	Utah Early Neuropathy Score (UENS) [42]	Early-stage detection	Sensitive measures for subtle change

To improve objectivity, examination-based scores such as the Neuropathy Disability Score (NDS) [40] and the Toronto Clinical Neuropathy Score (TCNS) [41] incorporate a structured assessment of vibration, reflexes, and pinprick sensation. These instruments offer greater reproducibility than symptom-only measures but tend to detect neuropathy only once significant deficits are present.

Comprehensive hybrid tools, most notably the Michigan Neuropathy Screening Instrument (MNSI) and Michigan Neuropathy Screening Test [42], attempt to combine the strengths of both approaches by integrating symptom questionnaires with physical examination. While this offers a broader clinical picture, their use requires examiner training and standardisation, which can limit feasibility in routine practice.

Other instruments focus more explicitly on functional outcomes. Function-oriented tools such as the Neuropathy Impairment Score (NIS) [43] quantify deficits across motor, sensory, and reflex domains and are frequently employed in clinical trials to monitor disease progression. Although they provide valuable longitudinal data, their sensitivity for early disease remains limited. More recently, scoring systems specifically designed to detect subtle changes have emerged, most notably the Utah Early Neuropathy Score (UENS) [44], which targets small fibre dysfunction in the feet and lower limbs. Early evidence suggests it may identify neuropathy at a stage when conventional tools remain normal, though its application is currently confined to research settings.

Despite the availability of numerous questionnaires, the lack of consensus on the most diagnostically accurate instrument continues to limit clinical translation. In recognition of this, several recent studies have compared the diagnostic performance of various screening tools. For example, the MNSI, with a cut-off score of >2, demonstrated a sensitivity of 96.8% and specificity of 85.7% for detecting DPN [45]. In comparison, the TCNS, at a cut-off of >2, exhibited a sensitivity of 73.2% and specificity of 64.0%, suggesting it is more prone to false positives but may detect more cases overall [46].

The NIS is noted for its very high specificity of 98.65%, but its sensitivity of 59.65% limits its ability to detect neuropathy in its early stages. This makes it more suited for confirming the presence of neuropathy once significant deficits have developed [45]. On the other hand, the UENS, specifically designed to detect early-stage neuropathy, showed a sensitivity of 91.7% and specificity of 83.3%, suggesting that the UENS may be more effective in identifying neuropathy during its earliest stages, when other tools remain normal [47].

While nerve conduction studies (NCS) are still considered the gold standard for diagnosing diabetic neuropathy, these questionnaires offer a valuable adjunct for screening, staging, and monitoring the disease, particularly in settings where time, resources, or specialist expertise may be limited. The choice of which questionnaire to use should depend on the clinical context, the specific population being screened, and the intended purpose of the assessment. These factors, along with examiner training and the need for standardisation, must also be considered to ensure the most accurate and useful results.

Nerve Conduction Studies

NCS remain the most established objective test for diabetic neuropathy and are widely considered the gold standard for diagnosing large fibre involvement. By assessing parameters such as conduction velocity, amplitude, and F-wave latency across motor and sensory nerves [48], NCS provide reproducible and quantifiable data that are invaluable for both clinical practice and research. Their objectivity and standardisation make them the benchmark against which newer diagnostic modalities are often evaluated [49-51]. They are also simple and non-invasive to conduct [52].

Despite these advantages, NCS are poorly suited to the early detection of DSPN. The technique primarily evaluates large myelinated fibres, while the earliest pathological changes in DSPN typically affect small Aδ and C fibres. As a result, patients may demonstrate normal NCS results even in the presence of significant small fibre dysfunction, contributing to underdiagnosis in the early stages of disease [53]. This limitation has prompted increasing recognition that a new reference standard, sensitive to small fibre pathology, is required for both clinical diagnosis and research outcomes. Until such a standard is established, NCS remain useful for confirming established neuropathy and for excluding alternative causes of neuropathic symptoms, but their role in early DSPN detection is limited.

Quantitative Sensory Testing (QST)

QST has been widely used to detect early small fibre dysfunction in DSPN in both research settings and routine practice. By measuring thresholds for vibration, pressure, and thermal sensation, it provides a non-invasive way of assessing both large and small fibre integrity. Abnormalities detected by mechanical testing (e.g., vibration perception threshold (VPT)) suggest large fibre damage, whereas thermal testing abnormalities (e.g., changes in warm perception threshold (WPT) and cold perception threshold (CPT)) suggest small fibre damage [54]. A recent position statement by the American Diabetes Association recommended testing temperature sensation for the detection of small fibre neuropathy (SFN) [6]. Since small fibre damage often precedes large fibre damage, VPT is not considered suitable for diagnosing early DSPN.

QST is painless, non-invasive, and well tolerated by patients [55], which makes it suitable for longitudinal monitoring. Thermal QST involves applying thermodes to the skin to determine the range of temperature perception. Two methods are commonly used: the method of limits [56], in which temperature is gradually changed until the patient signals detection, and the method of levels, in which set temperatures are applied and adjusted based on the patient’s responses [56].

Despite the advantages of QST, it has important limitations. Thermal QST is psychophysical: results depend on patient cooperation and can be influenced by attention, fatigue, anxiety [57], or communication difficulties [54]. External factors such as room temperature, background noise, and equipment variability further compromise reproducibility [58]. As a result, sensitivity varies greatly, from 36% to 85% [55]. Additionally, QST cannot differentiate between simulated and genuine sensory loss [59], limiting uptake in routine clinical practice.

Systematic reviews highlight inconsistencies in the methodology and overall quality of QST studies. Moloney NA et al. [56] assessed 21 thermal QST studies and found that none of the parameters showed consistent reliability. Among the studies focused on diabetic neuropathy, only one of the seven studies included in the review was considered high quality using the Quality Appraisal of Reliability Studies (QAREL) checklist. Half did not report whether environmental factors such as room temperature were controlled, despite this directly affecting the instruments. Three studies did not specify the type of device used, and blinding was unclear in all cases.

Recent initiatives have sought to address these shortcomings. The German Research Network on Neuropathic Pain developed a standardised protocol and normative dataset [54], demonstrating improved inter-observer and test-retest reliability when examiner training and certification were implemented [58]. Advances in portable technology, such as the NerveCheck device, may improve feasibility in clinical settings by offering a rapid, inexpensive assessment of multiple sensory modalities. Preliminary studies suggest diagnostic performance comparable to established QST systems; however, concerns regarding sensitivity and potential bias in industry-sponsored research remain.

The NerveCheck device, weighing 325 g, takes 3-13 minutes to complete testing depending on the number of modalities assessed [60]. An early study involving 130 participants, 74 with diabetes (type 1 and type 2) and 56 controls, compared NerveCheck with the neuropathy disability score and nerve conduction velocity tests [60]. VPT, WPT, and CPT all demonstrated high sensitivity; VPT demonstrated high specificity, while WPT and CPT demonstrated moderate specificity (81%, 67%, and 66%, respectively) [60]. The lower specificity of thermal thresholds may limit its usefulness for detecting small fibre damage. Although the authors concluded that NerveCheck has diagnostic accuracy comparable to established devices, the variability across QST studies and the weak reference standard must be considered.

A further study reported moderate sensitivity of both CPT and WPT when compared against intraepidermal nerve fibre density (IENFD) and corneal nerve fibre density, with CPT sensitivity as low as 53% against IENFD [61]. Specificity was better, ranging between 76% and 85%, and the authors concluded that NerveCheck has good diagnostic accuracy for SFN [61]. However, the low sensitivity limits its use as a stand-alone diagnostic tool, as many cases may be missed. Notably, the devices used in both studies were provided by the manufacturer, who also funded half of the research, authored both papers, and contributed to conference funding for the first study.

Overall, QST provides valuable insights into small fibre function and is particularly useful for longitudinal monitoring in clinical trials, where its non-invasive nature permits repeated measurements. Yet, variability in methodology, subjectivity, and limited reproducibility suggest it is unsuitable as a standalone diagnostic tool for early DSPN. Further standardisation and independent validation are required before QST can be reliably integrated into routine screening or diagnostic pathways.

Contact Heat Evoked Potentials

**Figure 1:**
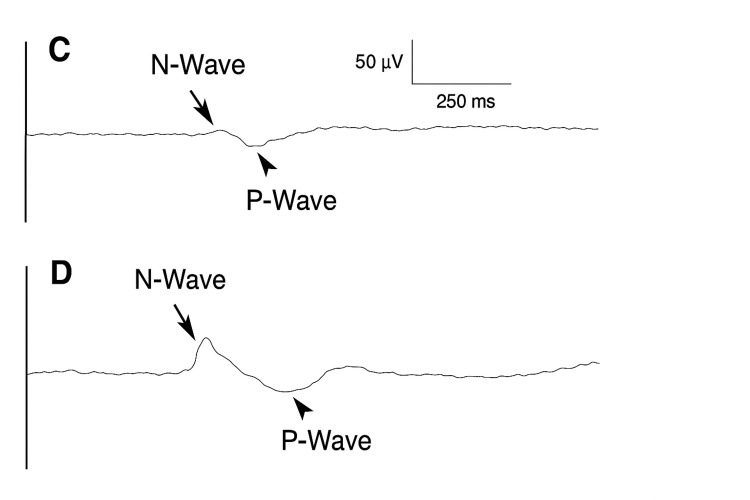
Average tracing of Contact Heat Evoked Potentials (CHEPs) in type 2 diabetic neuropathy. C shows the averaged CHEP tracing in a 60-year-old female patient with type 2 diabetic neuropathy. The N- and P-waves are reduced in amplitude and the N-wave demonstrates prolonged latency compared with the well-defined N- and P-waves shown in D. D is included for comparison and illustrates the averaged CHEP tracing in a 58-year-old healthy female. Adapted from reference [62]. CHEP: Contact Heat Evoked Potential.

Contact Heat Evoked Potentials (CHEPs) are an emerging technique for assessing small fibre function in diabetic neuropathy [62-64]. CHEPs involve rapid, selective thermal stimulation of Aδ and C fibres, producing cortical evoked potentials that can be objectively recorded from the scalp [63]. Results correlate significantly with IENFD from skin biopsies [62,65]. The typical negative-positive (N-P) waveform of CHEPs is mediated by small thermo-nociceptive fibres, and analysis of the tracing provides insight into SFN (Figure 1). Studies have consistently demonstrated reduced amplitudes and prolonged latencies of the N-P wave in patients with DPN compared with healthy subjects [62-64]. Moreover, CHEP responses may differentiate between positive and negative DPN symptoms, as amplitudes are higher in patients with painful DPN compared to those without symptoms. Therefore, CHEPs may be useful for evaluating treatment responses in DPN [62,63,66,67]. Unlike psychophysical tests such as QST, CHEPs provide quantifiable neurophysiological responses, making them less susceptible to subjectivity and patient bias.

Three key studies have demonstrated significant differences in CHEP responses between patients with DSPN and matched controls, with reduced amplitudes and prolonged latencies consistently observed. Chao CC et al. [62] administered contact heat to the dorsum of the foot and compared the resulting CHEP responses with IENFD. The sensitivity was high, with 81.3% of patients with DPN demonstrating reduced CHEP amplitudes or prolonged latencies. In an observational study, Wong MC and Chung JW [64] reported significant differences in CHEP responses between patients with DPN symptoms compared with both healthy controls and patients without symptoms. The TCNS [41] was used to quantify neuropathic symptoms and signs; however, it is recognised as having limited sensitivity compared with other tools. Notably, CHEP responses at the left lateral malleolus demonstrated greater sensitivity than those obtained from the dorsum of the foot, highlighting the importance of site selection in analysis.

Parson HK et al. [63] administered contact heat stimuli at six anatomical sites, reporting that the difference between control and DSPN patients was most pronounced at the lower back. On this basis, the authors concluded that the lower back may represent the optimal site for CHEP assessment, a finding supported by evidence suggesting spinal cord involvement in the early stages of DSPN [68]. However, at the lower leg site, only 20 of 30 patients responded to the stimuli, compared with 25 of 30 at the lower back, raising concerns about the reliability of this conclusion. This limitation reflects a broader challenge within CHEP studies [62-64], namely the frequent reliance on relatively small sample sizes, which may compromise the robustness of findings.

Although multiple studies have demonstrated the reproducibility of CHEPs [69,70], research specifically involving patients with T2DM remains limited. Across DPN cohorts, the sensitivity of CHEP appears high; however, future investigations should prioritise larger and more diverse populations that reflect the heterogeneity of routine clinical practice to improve generalisability. While normative data for CHEP have been published, the absence of a standardised methodology continues to restrict its clinical utility and hinders cross-study comparability [71].

Nerve and Skin Biopsy

Nerve biopsy has been used to diagnose early DSPN [25]; however, its clinical utility is limited due to its complexity. A nerve biopsy is an invasive procedure requiring specialist expertise, and analysis of nerve fibre density requires an electron microscope.

By contrast, skin biopsy is a minimally invasive method for quantitatively assessing IENFD [55]. The technique typically involves a 3-mm punch biopsy from the distal leg (requiring anaesthetic) [72]. More recently, blister biopsy using a suction capsule has emerged as a less invasive technique [72]. Reduced IENFD correlates with DSPN [73] and can be detected in individuals with impaired glucose tolerance (IGT) and recently diagnosed T2DM, suggesting high sensitivity [21]. International normative datasets have been developed for biopsies at the distal leg [74], strengthening its value as a research reference standard.

Despite these advantages, several practical limitations restrict the widespread use of skin biopsy. Calculating IENFD requires time-consuming histological processing [74] and access to specialist laboratories [19]. Repeat biopsies are often necessary for longitudinal studies, which some patients may find unacceptable due to the invasive nature of the procedure.

Reported sensitivity and specificity vary widely depending on the calculation method and reference standard (ranging from approximately 64% to 88%), reflecting the absence of consensus on diagnostic thresholds [75].

Some studies suggest that distal leg or ankle biopsies yield high diagnostic accuracy [24] and that skin biopsy is more sensitive than NCS and QST for early SFN [76]. Conversely, others have demonstrated normal IENFD in a subset of patients in whom QST had identified SFN [77]. These findings highlight both the strengths and limitations of skin biopsy and support a multimodal approach to diagnosis [78].

Overall, skin biopsy is generally more sensitive than NCS and QST for detecting early SFN and is less invasive than nerve biopsy. Nonetheless, its invasiveness, resource requirements, and limited accessibility make it impractical for routine clinical screening. For these reasons, attention has increasingly turned toward non-invasive alternatives such as corneal confocal microscopy (CCM).

Corneal Confocal Microscopy

CCM is increasingly recognised as one of the most promising non-invasive techniques for early detection of DSPN. The cornea has the highest density of small nerve fibres in the human body [79] and contains Aδ and C fibres [19]. Examination of Bowman’s layer provides a readily accessible window into small fibre pathology [79]. These corneal nerves have the same morphology as those affected in DSPN and may reflect similar damage; therefore, they may be used as a surrogate marker [24]. Using high-resolution laser scanning, CCM enables evaluation of several morphological features of corneal nerves: nerve fibre density (CNFD), nerve branch density (CNBD), nerve fibre length (CNFL), and nerve fibre tortuosity (CNFT) [80].

CCM offers several advantages over biopsy-based methods. It is non-invasive [24], well tolerated, and can be performed in an outpatient setting in less than ten minutes (five minutes per eye) [81]. Importantly, it is sensitive to early changes, with abnormalities detectable in individuals with IGT and recent-onset diabetes [82]. Longitudinal studies have shown that CCM can capture evidence of nerve repair following therapeutic interventions, positioning it not only as a diagnostic tool but also as a potential biomarker of treatment efficacy [83].

CCM allows in vivo visualisation of the corneal sub-basal nerve plexus. A laser scanning confocal microscope captures detailed images of the corneal layers, focusing on small unmyelinated nerve fibres within the sub-basal plexus. These images can be quantitatively analysed to assess nerve fibre density, length, and branching, critical metrics in the evaluation of SFN [84,85]. The procedure involves applying a topical anaesthetic and placing a lens on the cornea [80,86]. Studies demonstrate that CCM is safe and well tolerated across age groups, including children, with minimal discomfort reported [80,87]. Its non-invasive nature, absence of radiation, and short imaging duration (typically under ten minutes) contribute to its feasibility in paediatric settings [78,88]. Moreover, as no sedation or pupil dilation is required, it offers significant advantages over invasive procedures [87,89].

Normative datasets are now available across multiple populations [90], and standardised protocols have been published, strengthening the reproducibility of CCM. Unlike CHEPs, CCM already has an established protocol for early diabetic neuropathy, first outlined in 2011 [80].

Tavakoli M et al. [91] demonstrated that increasing DPN severity, assessed by neuropathy deficit scores, correlated with greater corneal nerve degeneration on CCM. Notably, nerve fibre damage was detected in patients with a neuropathy deficit score of 0, suggesting that CCM may be more sensitive than clinical examination for early DPN detection. Similarly, Chen X et al. [92] reported that CCM was more sensitive than the current gold standard IENFD, although the difference did not reach statistical significance.

Ziegler D et al. [21] found that CCM detected nerve fibre loss more frequently than skin biopsy; however, discordance was common, with some patients showing normal CNFD but abnormal IENFD and vice versa, a phenomenon also reported with other diagnostic tests [93]. This may reflect the heterogeneous and asynchronous nature of SFN. Methodological differences may also contribute: Ziegler D et al. [21] used a longer and more detailed imaging protocol (15 minutes) than most other CCM studies [5,80,94-96,81-83,86,88,90,91,93]. Moreover, divergent results between CNFD and IENFD raise the possibility that the cornea, which lacks vasculature, may not fully capture the vascular-neuropathy interplay underlying peripheral SFN [5]. Given that SFN may develop asynchronously across organ systems, corneal nerve loss may represent a distinct but related pathological process [96]. Nonetheless, improved understanding of DPN pathophysiology remains essential to ensure surrogate markers are reliable [24]. Current guidelines therefore recommend a multimodal diagnostic approach [58]. Compared with nerve biopsies and CHEPs, CCM presents fewer limitations, and recent longitudinal data confirm progressive loss of CNFL in DPN patients [97]. Collectively, these findings highlight CCM as a promising modality for early and sensitive DPN detection.

CCM may require optometrist or ophthalmologist expertise to perform [90]. Therefore, integrating CCM into multidisciplinary diabetes care may involve alignment with diabetic retinopathy screening programmes where appropriately skilled professionals are available. CCM may be particularly useful for patients with confirmed diabetes, for whom screening is recommended annually [98], as it is non-invasive and appropriate for both adults and children [95]. This was clearly illustrated in a study comparing skin biopsy and CCM for small fibre quantification in DSPN diagnosis; recruitment was limited by participants declining biopsy, highlighting the greater acceptability of CCM.

Concerns regarding the reproducibility of CCM, particularly the subjectivity involved in assessing CNBD, have prompted the development of automated algorithms for SFN analysis [94] to improve inter-rater reliability. Petropoulos IN et al. [81] evaluated CCM images from 186 patients with diabetes and 55 controls using both manual and automated quantification. Strong correlations were observed across CNFL, CNFD, and CNBD, with the automated system demonstrating excellent reproducibility on retesting. Notably, automated analysis was significantly faster, requiring 10-22 seconds compared with 2-7 minutes for manual assessment [81], underscoring its potential to streamline routine clinical evaluations and support larger-scale clinical trials.

The integration of AI with CCM offers further promise in enhancing diagnostic accuracy, efficiency, and scalability. Manual analysis is time-intensive and subject to inter-observer variability, limiting clinical utility. In contrast, AI models, particularly deep learning and convolutional neural networks (CNNs), have demonstrated accurate and reproducible segmentation and quantification of corneal nerve fibres [94,99]. These approaches enable rapid, objective assessment of nerve fibre density, length, and branching, critical biomarkers in neuropathic conditions such as diabetes [99,100]. AI-enhanced CCM may therefore enable earlier detection of subclinical neuropathy, longitudinal tracking of disease progression, and evaluation of therapeutic response, strengthening its value as a diagnostic and research tool [101].

Sudorimetry

Sweat glands are innervated by small unmyelinated C fibres, and the evaluation of sweating therefore provides a means of quantifying small fibre function [102]. Dysfunction occurs early in diabetes and contributes to foot ulcer risk [103] through reduced sweating, which leads to skin dryness, fissuring, and infection [104].

Neuropad is an adhesive patch that changes colour from blue to pink in response to sweat, thereby providing an indirect measure of skin hydration [105]. The rate of colour change is proportional to the degree of skin hydration [105]. It is easy to use and suitable for patient self-management, with good concordance between patient and physician assessment [106]. A meta-analysis reported a modest specificity of 65% but a higher sensitivity of 86% [107]. This limits Neuropad as a stand-alone diagnostic tool but supports its use as a screening or triage method. Although its specificity has been criticised, its speed and simplicity may outweigh this limitation. Longitudinal data suggest Neuropad may predict the risk of neuropathy, as patients with abnormal baseline results were more likely to develop neuropathy at five years [108], and Neuropad has also shown utility in pre-diabetes screening [109]. This is clinically relevant since individuals at risk of type 2 diabetes are not routinely screened, yet often present with neuropathy. However, the cost-effectiveness of widespread early screening remains unclear, given the limited evidence for reversal of DSPN.

The Sudoscan is a rapid (less than five minutes), non-invasive and inexpensive method of measuring electrochemical skin conductance in the hands and feet via the chloride component of sweat [110]. It has demonstrated high sensitivity for SFN, with Smith AG et al. [84] reporting 77% sensitivity and 67% specificity using the UENS. Casellini CM et al. [110] observed comparable sensitivity but higher specificity (92%) using the NIS. Both studies involved diverse patient cohorts, enhancing generalisability. However, Smith AG et al. [93] reported lower specificity, likely due to sex-related differences in sweating, since females (who represented a higher proportion in this study) may produce less sweat [111,112]. Furthermore, most participants were Caucasian, yet sudomotor function varies across populations [113], indicating the need for more ethnically diverse and gender-balanced cohorts. Importantly, neither study employed skin biopsy, the current gold standard for detecting SFN, because of its invasiveness. Instead, composite symptom and sign scoring systems were used, which may be less sensitive and reliable [59]. Methodological differences across studies further complicate comparisons, underscoring the need for standardised protocols in sudomotor testing.

Quantitative Sudomotor Axon Reflex Testing (QSART) and autonomic function testing (AFT) are valuable for assessing small fibre involvement in diabetic neuropathy. Recent studies suggest that laser-evoked potentials may also be useful in early detection [114], although these methods are less widely available in routine clinical practice. A summary of commonly used diagnostic tests for small and large fibre neuropathy is shown in Table 4.

**Table 4:** Summary of diagnostic tests for diabetic neuropathy.

Diagnostic Test	Fibre Type Assessed	Key Features	Advantages	Limitations
Monofilament (10 g)	Large fibres	Pressure sensation	Quick, inexpensive, widely used	Detects only advanced neuropathy; insensitive to early/small fibre damage
Vibration Perception Threshold (VPT)	Large fibres	Vibration sense	Objective, easy to perform	Less sensitive to early/small fibre dysfunction
Nerve Conduction Studies (NCS)	Large fibres	Motor and sensory conduction velocity	Gold standard for large fibre neuropathy	Fails to detect early small fibre damage
Quantitative Sensory Testing (QST)	Small and large fibres	Temperature, pain, vibration thresholds	Can detect small fibre dysfunction	Requires patient cooperation; psychophysical/subjective
Corneal Confocal Microscopy (CCM)	Small fibres	Corneal nerve density and morphology	Non-invasive; detects early small fibre loss	Requires specialised equipment; limited availability
QSART (Quantitative Sudomotor Axon Reflex Test)	Small fibres (autonomic)	Measures sudomotor function	Detects autonomic small fibre dysfunction	Specialised test; limited availability
Autonomic Function Testing (AFT)	Small fibres (autonomic)	Heart rate variability, BP responses	Detects early autonomic involvement	Requires training; time-consuming
Laser-Evoked Potentials (LEP)	Small fibres	Nociceptive fibre function	Sensitive for early small fibre damage	Specialised equipment; limited clinical availability

## Conclusions

The pathophysiology of DPN is complex, and current understanding is limited by the use of surrogate endpoints, which lack reliability, as well as issues with patient selection in research studies. Debate persists regarding the underlying mechanisms and diagnostic criteria for DSPN, and clearer insights are needed to determine whether the techniques discussed in this review are optimal for early detection. Normal findings in highly sensitive tests do not exclude DSPN, and combining multiple diagnostic modalities may improve clinical accuracy. Development of guidelines for the sequential use of tests that detect SFN could replace traditional approaches that fail to capture early disease. Longitudinal, prospective studies are essential to validate diagnostic methods for early DSPN. Comparing diagnostic accuracy remains challenging due to the absence of a consistent reference standard for small fibre damage; nerve conduction studies and composite neuropathy scores are commonly used but primarily reflect large fibre dysfunction and may overlook early changes. Furthermore, the lack of methodological standardisation across studies complicates interpretation, while larger trials often include heterogeneous causes of small fibre neuropathy, highlighting the need for studies focused specifically on DSPN.

Non-invasive diagnostic tools such as Sudoscan and CCM, which have been evaluated across diverse diabetic cohorts, show particular promise for detecting early DPN. The availability of established protocols and normative data for CCM supports its clinical translation, although its requirement for specialised equipment may limit accessibility. Further studies are needed to assess its generalisability in routine practice. Advances in automated corneal nerve quantification offer opportunities to enhance diagnostic speed and reproducibility, both of which are critical for clinical care and research.
